# Protective Effects of Eugenol Against Monosodium Glutamate-Induced Reproductive Toxicity in Male Wistar Rats

**DOI:** 10.3390/jox16010033

**Published:** 2026-02-13

**Authors:** Kouthulgama Veekshith Reddy, Majid Shafi, Abhinav Madari, Sharath Chandra Goud, Shayaib Ahmad Kamil, Akeel Bashir, Masood Saleem Mir, Mir Nadeem Hassan, Mudasir Ali Rather, Zahoor Ahmad Wani, Showkeen Muzamil Bashir, Atif Khurshid Wani, Showkat Ahmad Shah

**Affiliations:** 1 Division of Veterinary Pathology, Faculty of Veterinary Sciences and Animal Husbandry, Sher-e-Kashmir University of Agricultural Sciences and Technology of Kashmir, Srinagar 190006, Jammu and Kashmir, India; vikkyreddy.92@gmail.com (K.V.R.); majidkawoosa@skuastkashmir.ac.in (M.S.); madariabhinav@gmail.com (A.M.); sharathcg@gmail.com (S.C.G.); kamilshoaib@gmail.com (S.A.K.);; 2 Division of Veterinary Microbiology & Immunology, Faculty of Veterinary Sciences and Animal Husbandry, Sher-e-Kashmir University of Agricultural Sciences and Technology of Kashmir, Srinagar 190006, Jammu and Kashmir, India; 3 Division of Veterinary Public Health, Faculty of Veterinary Sciences and Animal Husbandry, Sher-e-Kashmir University of Agricultural Sciences and Technology of Kashmir, Srinagar 190006, Jammu and Kashmir, India; 4 Division of Veterinary Parasitology, Faculty of Veterinary Sciences and Animal Husbandry, Sher-e-Kashmir University of Agricultural Sciences and Technology of Kashmir, Srinagar 190006, Jammu and Kashmir, India; 5 Division of Veterinary Biochemistry, Faculty of Veterinary Sciences and Animal Husbandry, Sher-e-Kashmir University of Agricultural Sciences and Technology of Kashmir, Srinagar 190006, Jammu and Kashmir, India

**Keywords:** monosodium glutamate (MSG), eugenol, reproductive toxicity, oxidative stress, Wistar rats

## Abstract

Monosodium glutamate (MSG), a widely used flavor enhancer, has been implicated in oxidative stress-mediated systemic and reproductive toxicity, particularly affecting the male gonadal system. This study evaluated the ameliorative potential of eugenol, a phenolic compound with potent antioxidant properties, against MSG-induced reproductive toxicity in male Wistar rats. Thirty rats were randomly divided into five groups: group 1 (control), group 2 (MSG 2.5 g/kg), group 3 (eugenol 200 mg/kg), group 4 (MSG + eugenol 100 mg/kg), and group 5 (MSG + eugenol 200 mg/kg). Treatments were administered orally for 28 days. Hematological, biochemical, hormonal, antioxidant, gross, and histopathological assessments were conducted after sacrifice on Day 29. MSG exposure significantly reduced testicular weight, testosterone levels, TEC, Hb, PCV, serum proteins, and testicular GSH and SOD, while markedly elevating TLC, AST, ALT, BUN, creatinine, and TBARS. Severe testicular degeneration, vascular congestion, germ-cell loss, and disrupted seminiferous tubules were observed histologically. Co-administration of eugenol resulted in significant and dose-dependent amelioration of MSG-induced alterations, restoring hematological and biochemical parameters, improving antioxidant status, and elevating testosterone levels. Gross pathology and histopathology demonstrated progressive structural recovery, with the higher eugenol dose showing near-normal testicular architecture and active spermatogenesis. Eugenol alone produced no adverse effects and remained comparable to the control group across all parameters. The findings indicate that eugenol confers strong protective effects against MSG-induced reproductive toxicity, primarily through its antioxidant and cytoprotective actions. Eugenol may serve as a promising natural therapeutic agent for mitigating chemically induced male reproductive impairments.

## 1. Introduction

Food additives are chemical substances deliberately added to food to enhance its flavor, texture, color, shelf life, and overall acceptability. These additives include preservatives, antioxidants, stabilizers, colorants, and flavor enhancers, each serving specific technical purposes in food processing [1]. Among them, flavor enhancers are widely used, and glutamate salts, particularly monosodium glutamate (MSG), are the most common [2]. MSG was first isolated in 1908 by Kikunae Ikeda and is responsible for imparting the unique umami taste, which is now considered the fifth basic taste alongside sweet, sour, salty, and bitter [3]. Commercially, MSG is produced through microbial fermentation of carbohydrate-rich materials such as molasses, sugarcane, and corn starch using *Corynebacterium glutamicum* [4]. Although MSG is widely used and recognized as safe by the United States Food and Drug Administration (USFDA), several studies have raised concerns about its long-term and high-dose consumption. Excessive MSG intake has been linked to “Chinese Restaurant Syndrome” (CRS), characterized by headache, flushing, muscle tightness, and weakness [5]. Furthermore, MSG has been associated with neurotoxicity, obesity, metabolic disorders, and systemic organ damage [6]. High dosages of MSG can affect several biological systems, but the reproductive system is thought to be its main target [7]. This is because seminiferous tubules and spermatozoa have low levels of antioxidant reserve and an abundance of glutamate receptors and polyunsaturated fatty acids, which makes them more vulnerable to peroxidative and excitatory damage [8]. Prolonged exposure has been reported to induce testicular degeneration, decreased sperm count, abnormal sperm morphology, oligozoospermia, and reduced testosterone levels, all of which may contribute to infertility [9,10]. There are still many disagreements regarding its testicular toxicity in relation to variations in the age of the experimental model, the amount of MSG administered, and the length of exposure, which motivates further investigation into the multifactorial nature of testicular impairment. The primary causes of MSG’s acceleration of apoptotic cell death are an increase in intracellular calcium concentration and a disruption in cellular redox potential brought on by activation of the Krebs cycle [11]. By interfering with the hormonal controlling mechanisms, its suppressive action on the hypothalamic–pituitary–gonadal axis adds another dimension. It can cause germ cells to undergo apoptosis and has detrimental effects on the oxidative state and histoarchitecture of the testis [2,7].

These findings also highlight the need to investigate protective agents capable of mitigating MSG-induced oxidative and reproductive damage. In this regard, natural bioactive compounds from spices and medicinal plants have gained significant attention. Spices such as turmeric, cinnamon, ginger, and clove contain phenolic compounds, terpenes, and flavonoids with strong antioxidant, anti-inflammatory, antimicrobial, and anticancer properties. Among these, eugenol (4-allyl-2-methoxyphenol) is a phenylpropanoid found abundantly in clove (*Eugenia caryophyllata*) and cinnamon (*Cinnamomum verum*), constituting 45–90% and 20–50% of their essential oil content, respectively [12]. Eugenol has been reported to possess multiple pharmacological activities, including antioxidant, anti-inflammatory, analgesic, anti-allergic, antimicrobial, and anticancer effects [13]. Eugenol is declared as GRAS (generally recognized as safe) by the World Health Organization (WHO) and is considered non-mutagenic. Eugenol is readily absorbed following oral administration and undergoes extensive hepatic metabolism through phase I (oxidation) and phase II (glucuronidation and sulfation) pathways, resulting in water-soluble metabolites that are efficiently excreted [12,13]. The biological activity of eugenol is primarily attributed to its potent antioxidant and cytoprotective properties, including direct scavenging of reactive oxygen species, inhibition of lipid peroxidation, and chelation of redox-active metal ions [14,15]. In addition, eugenol enhances endogenous antioxidant defenses by modulating enzymes such as superoxide dismutase and glutathione-dependent systems and suppressing oxidative stress-induced inflammatory and apoptotic signaling [16,17]. The antioxidant and free radical scavenging properties of eugenol are well-documented [15,18]. By creating complexes with decreased metals, eugenol’s antioxidant capacity can be clarified. Isoeugenol and eugenol have a strong inhibitory effect on lipid peroxidation because they eliminate free radicals and produce an iron–oxygen chelate complex by maintaining iron and copper in reduced states, respectively [14]. Therefore, the present study was undertaken with the aim of evaluating the ameliorative role of eugenol on MSG-induced male reproductive toxicity in Wistar rats.

## 2. Materials and Methods

### 2.1. Experimental Animals

A total of 30 male albino Wistar rats, aged 6–8 weeks and weighing 150–200 g, were procured from IIIM, Jammu. Animals were housed in polypropylene cages under a controlled 12 h light/dark cycle, 20–24 °C, and 30–70% RH, with sterile wood dust bedding, a pellet diet, and distilled water ad libitum. Animals were acclimatized for one week and observed thrice daily for clinical signs or mortality.

### 2.2. Chemicals

Analytical-grade chemicals and reagents were used. Monosodium glutamate was procured from HI Media, and Eugenol from Sigma-Aldrich (Saint Louis, MO, USA).

### 2.3. Experimental Design

Animals were randomly divided into five groups: Group 1: Control (normal saline); Group 2: MSG 2.5 g/kg; Group 3: Eugenol 200 mg/kg; Group 4: MSG 2.5 g/kg + Eugenol 100 mg/kg; and Group 5: MSG 2.5 g/kg + Eugenol 200 mg/kg. All treatments were given orally for 28 days. Monosodium glutamate (MSG) was first dissolved in normal saline prior to administration, whereas eugenol, owing to its oily nature, was administered in its native form. Oral gavage provides a physiologically relevant exposure route that closely mimics human dietary consumption while allowing accurate dose control and reproducibility. Furthermore, according to data available in the NCBI database, the lethal dose (LD_50_) of MSG was established in experimental animals. Based on these reports, a sub-lethal dose of 2.5 g/kg body weight was selected for the present animal experiment. Dose selection was based on LD50 values: MSG 16,600 mg/kg (oral, rat) and eugenol 1930 mg/kg (oral, rat) per NCBI (2024) [19].

### 2.4. Body Weight

The individual body weights of all the rats were measured on the first day and, consequently, on the 7th, 14th, 21st, and 28th day of the experiment to assess the weight changes.

### 2.5. Hematology

On the 29th day of the experiment, all six rats from each group were sacrificed. Prior to blood collection, the animals were fasted for 12 h. Before euthanasia, the rats were anesthetized, and 1 mL of blood was collected from the retro-orbital plexus using a 3 mm capillary tube. The blood was transferred into anticoagulant-coated vacutainers (K3-EDTA tubes, 13 mm × 75 mm, 6 mL; Rapid Diagnostics Pvt. Ltd., New Delhi, India). The collected blood samples were utilized to evaluate hematological parameters, including total erythrocyte count (TEC), hemoglobin concentration (Hb), packed cell volume (PCV), and total leukocyte count (TLC). These measurements were conducted by using an automated hematology analyzer (Nihon Kohden, Tokyo, Japan), ensuring precision and reliability.

### 2.6. Serum Biochemistry

Approximately 2 mL of blood was collected into non-EDTA vacutainers and left undisturbed in an inclined position for 1 h to allow serum separation. The serum was then transferred into Eppendorf tubes and stored at −20 °C for biochemical analyses. The following biochemical parameters were assessed: Albumin (ALB) (g/dL), total protein (TP) (g/dL), creatinine (mg/dL), blood urea nitrogen (BUN) (mg/dL), alanine aminotransferase (ALT) (IU/L), and aspartate aminotransferase (AST) (IU/L). These measurements were performed by using a semi-automatic serum analyzer (Robert Riele GmbH & Co KG, Berlin, Germany) to ensure accuracy and consistency.

### 2.7. Hormone Estimation

Testosterone content was evaluated by a hormonal assay using serum samples obtained from rats of every experimental group (*n* = 6). The samples were labeled and stored at −20 °C until further analysis. Testosterone concentrations were measured using a commercially available ELISA Kit (Rat Testosterone (T) ELISA kit—CUSABIO Cat No: CSB-E05100r, Wuhan, China), ensuring precise and efficient estimation. The assay was performed according to the manufacturer’s protocol.

### 2.8. Absolute Organ Weight

After the animals were sacrificed, the testes from all 30 animals were carefully collected. Each organ was cleaned with filter paper, and its weight was measured using an electronic balance to determine any changes in absolute organ weights.

### 2.9. Tissue Antioxidant Profile (Testis)

After sacrifice, 600 mg of testicular tissue was collected, homogenized, and stored at −20 °C to study the organ oxidative stress parameters like reduced glutathione (GSH), superoxide dismutase (SOD), and thio-barbituric acid reactive substances (TBARS) using standardized spectrophotometric methods [20,21,22].

For one gram of tissue sample (quantity of buffer was adjusted based on the weight of tissue to get 10 percent homogenate), 10 mL of 0.2 M Tris HCl buffer (pH 7.2) was added and homogenized to get a 10 percent solution of tissue and buffer for estimation of all the tissue oxidative stress parameters. The oxidation of tissue was measured by the reaction of the lipid peroxidation (LPO) end products, such as malondialdehyde (MDA), with thio-barbituric acid. The activity of reduced glutathione (GSH) and superoxide dismutase (SOD) was measured to determine the antioxidant status of the tissue.

### 2.10. Gross Pathology

Following blood collection, the rats were euthanized by cervical dislocation, and a detailed postmortem examination was conducted. Any observable gross lesions in the testes were carefully documented, and the organs were immediately weighed using an electronic balance.

### 2.11. Histopathology

The representative tissue samples of the testes from different groups were collected and fixed in 10% formalin for 24 h, followed by processing for histopathological examination. The tissue samples, approximately 0.5 × 0.5 cm in size, were dehydrated, cleared, and embedded in wax. The tissue sections were stained with Harris hematoxylin and eosin for routine examination following Luna’s protocol [23].

### 2.12. Statistical Analysis

The data were statistically analyzed by using a one-way Analysis of Variance (ANOVA) in Graph Pad Prism 5 (version 5.01, GraphPad Software, San Diego, CA, USA). Tukey’s test was applied to determine differences between means, with statistical significance set at *p* < 0.05 [24].

### 2.13. Ethical Approval

The study was conducted in accordance with the Declaration of Helsinki and approved by the Institute Animal Ethics Committee (IAEC) vide No. FVSc/IAEC/2025/44, dated: 10 November 2025.

## 3. Results

### 3.1. Body Weight

All the groups showed a consistent increase in body weight throughout the treatment period. Overall weight gain was greater in group II than in the control group, whereas Groups 3, 4, and 5 maintained patterns comparable to, or slightly higher than, the control group (Table 1).

### 3.2. Organ Weight

**Figure 1:**
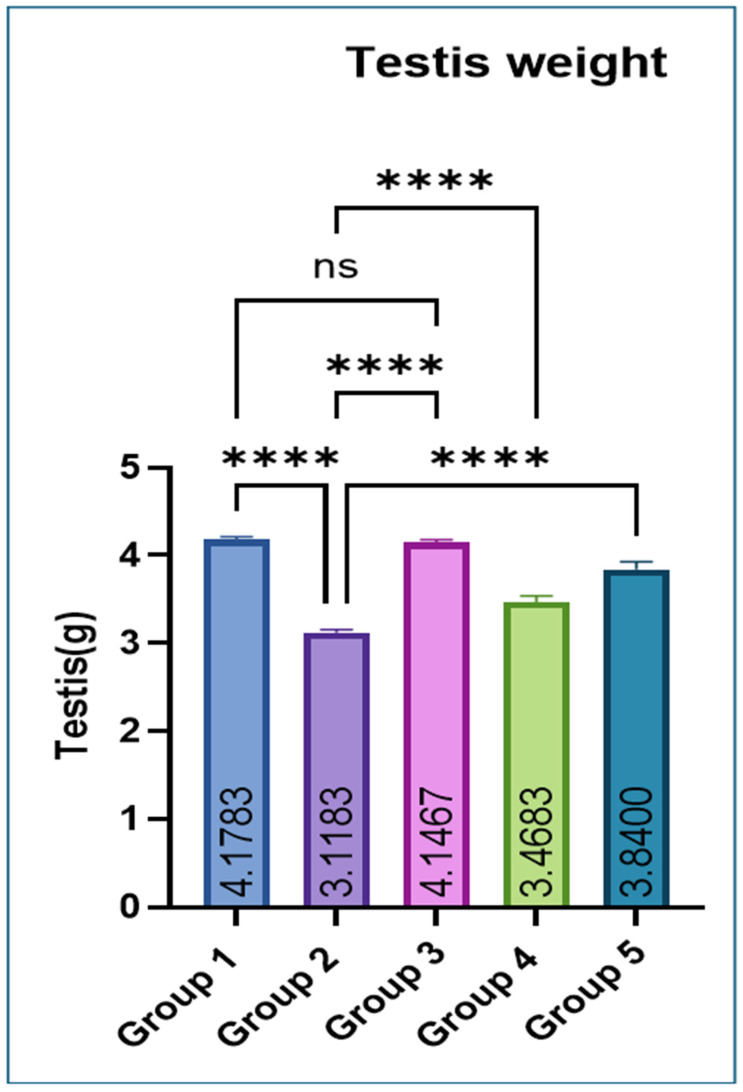
Effect of monosodium glutamate (MSG) and eugenol on absolute testicular weight in male Wistar rats. Group 1: Control (normal saline); Group 2: MSG (2.5 g/kg b.w.); Group 3: Eugenol (200 mg/kg b.w.); Group 4: MSG (2.5 g/kg b.w.) + Eugenol (100 mg/kg b.w.); Group 5: MSG (2.5 g/kg b.w.) + Eugenol (200 mg/kg b.w.). Values are expressed as Mean ± SE (*n* = 6). Data were analyzed using one-way ANOVA followed by Tukey’s multiple comparison test. * Asterisks indicate statistical significance compared with the MSG-treated group (Group 2): ****: *p* < 0.0001. “ns” means there is non-significant difference in the groups that are being compared.

Testicular weight was significantly lower in Group 2 compared with Groups 1 and 3. Co-administration of eugenol resulted in a significant improvement in testicular weight in Groups 4 and 5 (Figure 1).

### 3.3. Hematological Parameters

**Figure 2:**
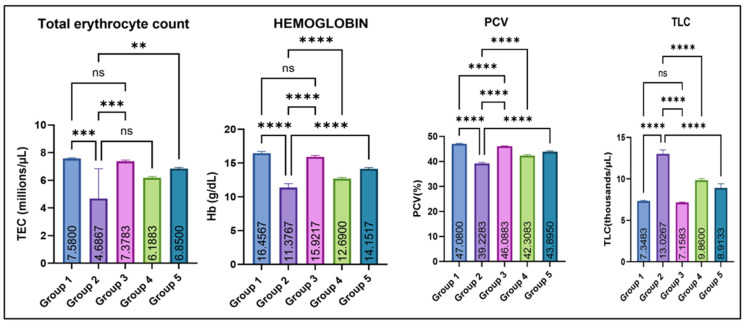
Effect of monosodium glutamate (MSG) and eugenol on hematological parameters (TEC, Hb, PCV, and TLC) in male Wistar rats on Day 29. Group 1: Control (normal saline); Group 2: MSG (2.5 g/kg b.w.); Group 3: Eugenol (200 mg/kg b.w.); Group 4: MSG (2.5 g/kg b.w.) + Eugenol (100 mg/kg b.w.); Group 5: MSG (2.5 g/kg b.w.) + Eugenol (200 mg/kg b.w.). Values are expressed as Mean ± SE (*n* = 6). Data were analyzed using one-way ANOVA followed by Tukey’s multiple comparison test. Asterisks indicate statistical significance compared with the MSG-treated group (Group 2): **: *p* < 0.01, ***: *p* < 0.001, and ****: *p* < 0.0001. “ns” means there is non-significant difference in the groups that are being compared.

As illustrated in Figure 2, Group 2 showed a significant decrease in mean total erythrocyte count (TEC, millions/µL), hemoglobin concentration (Hb-g/dL), and mean packed cell volume (PCV/hematocrit-%) as compared with the control (group I). Amelioration with eugenol (Groups 4 and 5) resulted in a marked improvement in these hematological parameters, while Group 3 (eugenol alone) remained comparable to the control. In contrast, total leucocyte count (TLC, thousands/µL) was significantly elevated in Group 2 compared with all other groups. Eugenol supplementation lowered TLC toward normal, while Group 3 remained comparable to the control.

#### 3.3.1. Serum Biochemical Values

**Figure 3:**
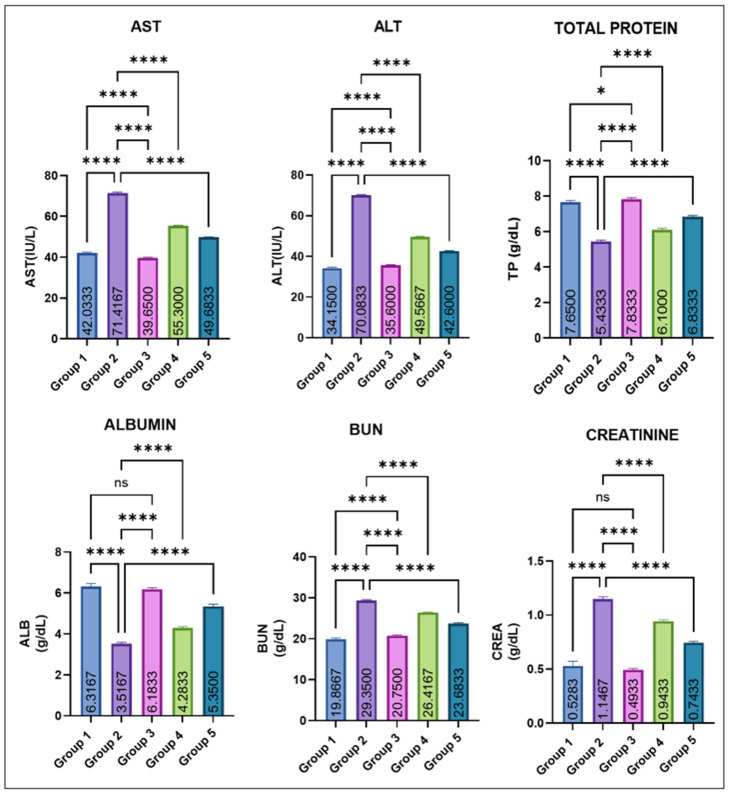
Effect of monosodium glutamate (MSG) and eugenol on serum biochemical parameters (AST, ALT, total protein, albumin, BUN, and creatinine) in male Wistar rats on Day 29. Group 1: Control; Group 2 MSG (2.5 g/kg); Group 3: Eugenol (200 mg/kg); Group 4: MSG + Eugenol (100 mg/kg); Group 5: MSG + Eugenol (200 mg/kg). Data are expressed as Mean ± SE (*n* = 6) and analyzed using one-way ANOVA followed by Tukey’s multiple comparison test. ** Asterisks indicate statistical significance compared with Group 2 (MSG): *: *p* < 0.05; **: *p* < 0.01; ***: *p* < 0.001; ****: *p* < 0.0001. “ns” means there is non-significant difference in the groups that are being compared.

The variations in various serum biochemical markers are shown in Figure 3. Group 2 showed a marked elevation in mean AST and ALT levels compared with all other groups. Eugenol administration resulted in a significant dose-dependent decrease in ALT and AST levels (groups 4 and 5), while groups 1 and 2 remained comparable to control. Group 2 recorded a significant fall in total protein (TP) compared with groups 1 and 3. Eugenol supplementation (groups IV and V) caused a progressive improvement, though still slightly lower than the control values. Serum albumin levels were also markedly decreased in group 2. Groups IV and V showed a significant recovery compared with group 2, yet remained modestly below the control and eugenol-only groups, which did not differ from each other. BUN and creatinine concentrations were significantly increased in group 2. Values in groups 4 and 5 were significantly lower than in Group 2 but still exceeded those of the control and Group 3. Groups 1 and 3 again remained statistically similar.

#### 3.3.2. Testosterone Concentration

**Figure 4:**
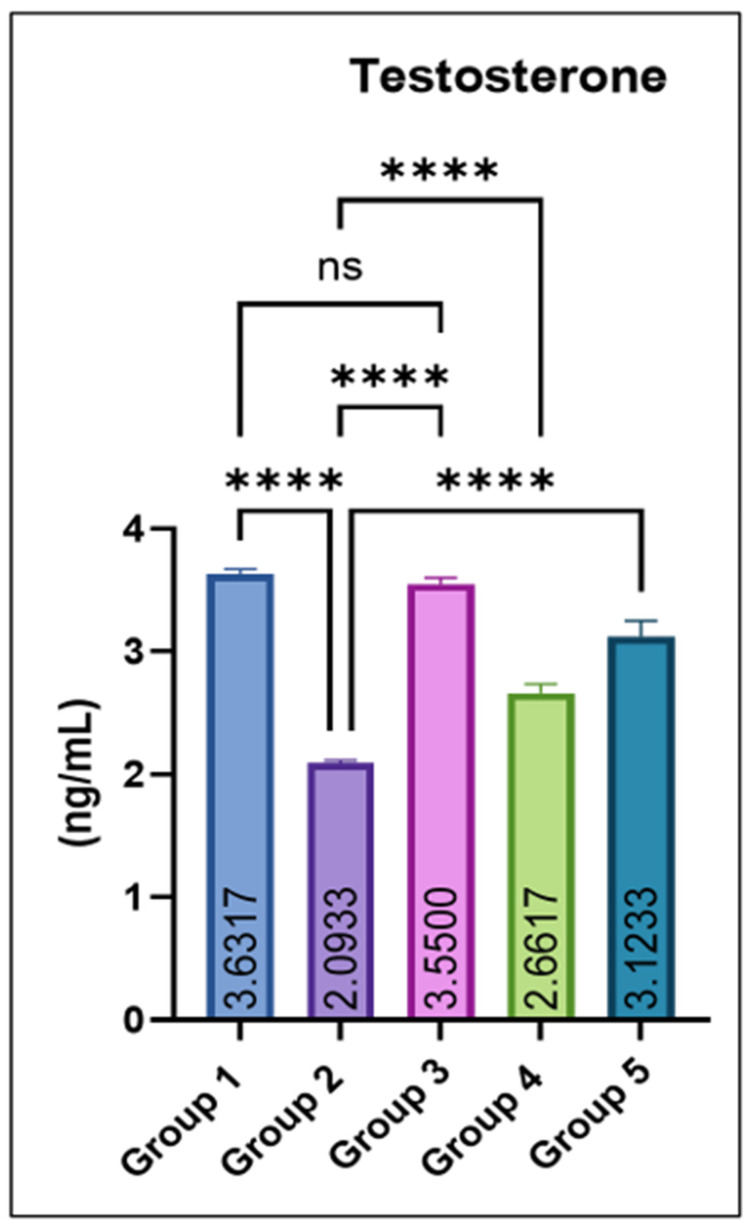
Effect of monosodium glutamate (MSG) and eugenol on serum testosterone concentration (ng/mL) in male Wistar rats. Group 1: Control; Group 2: MSG (2.5 g/kg); Group 3: Eugenol (200 mg/kg); Group 4: MSG + Eugenol (100 mg/kg); Group 5: MSG + Eugenol (200 mg/kg). Values are shown as Mean ± SE (*n* = 6). Statistical analysis was performed using one-way ANOVA followed by Tukey’s test. * Asterisks indicate statistical significance compared with the MSG-treated group (Group 2): ****: *p* < 0.0001. “ns” means there is non-significant difference in the groups that are being compared.

Serum testosterone concentrations were significantly reduced in group 2 compared with the control. Co-administration of eugenol produced a dose-dependent recovery, with groups 4 and 5 showing significant increases relative to group 2. The eugenol-only group (Group 3) maintained testosterone levels comparable to the control, as shown in Figure 4.

#### 3.3.3. Tissue Antioxidant Profile (Testis)

**Figure 5:**
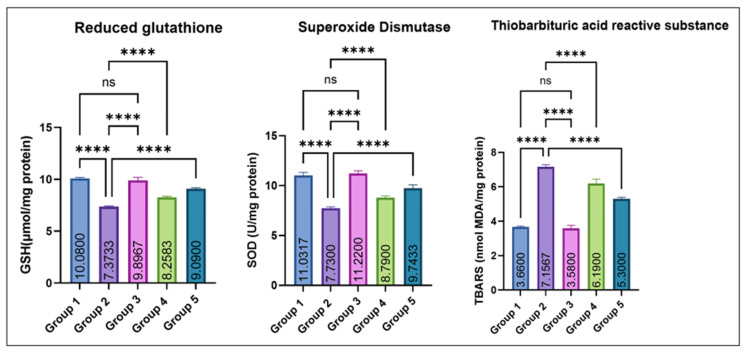
Effect of monosodium glutamate (MSG) and eugenol on testicular antioxidant parameters (GSH, SOD, and TBARS) in male Wistar rats. Group 1: Control; Group 2: MSG (2.5 g/kg); Group 3: Eugenol (200 mg/kg); Group 4: MSG + Eugenol (100 mg/kg); Group 5: MSG + Eugenol (200 mg/kg). Data are expressed as Mean ± SE (*n* = 6) and analyzed using one-way ANOVA followed by Tukey’s post hoc test. ** Asterisks indicate statistical significance compared with Group 2 (MSG): ****: *p* < 0.0001. “ns” means there is non-significant difference in the groups that are being compared.

As depicted in Figure 5, testicular GSH and SOD levels were significantly decreased in group 2 as compared to the control. Groups 4 and 5 exhibited a dose-dependent recovery, while Group 3 (eugenol alone) remained comparable to the control. No significant difference was noted between Groups 1 and 3. Conversely, TBARS concentrations were significantly elevated in Group 2 compared with the control, while co-treatment with eugenol resulted in a significant reduction in Groups 4 and 5; however, TBARS levels in these groups remained higher than the control, indicating partial but not complete attenuation of MSG-induced lipid peroxidation. Groups 1 and 3 showed no significant difference.

#### 3.3.4. Gross Pathology

**Figure 6:**
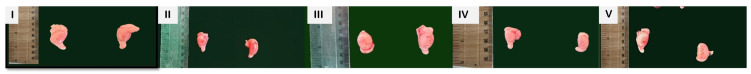
Gross pathological appearance of testes from different experimental groups on Day 29. Group 1 (control) and Group 3 (eugenol alone) show normal size and appearance. Group 2 (MSG-treated) shows congestion and reduced size. Groups 4 and 5 (MSG + Eugenol) show dose-dependent improvement toward normal morphology, with Group 5 exhibiting near-normal gross appearance.

On the 29th day of the experiment, testes were examined for gross changes (Figure 6). Groups 1 and 3 exhibited normal size and appearance, with no visible abnormalities. Group 2 (MSG only) showed severe congestion and a slight reduction in size, indicating marked testicular damage. Co-treatment with eugenol led to a progressive improvement as group 4 displayed mild congestion with near-normal size, while group 5 showed further restoration of normal size and only slight residual congestion.

### 3.4. Histopathology

**Figure 7:**
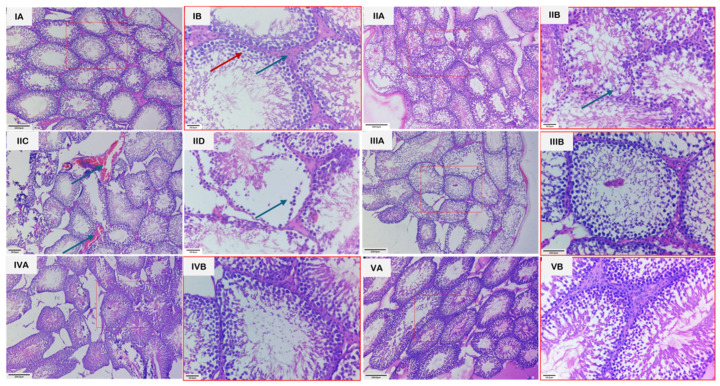
Photomicrographs of H&E-stained sections of testes from various groups. (**IA**): Section of testis from group 1 showing seminiferous tubules exhibiting normal architecture with active spermatogenesis, along with interstitial spaces containing Leydig cells. Scale bar length: 200 µm. (**IB**): Higher magnification of the same section, revealing spermatocytes at various stages of development (red arrow) and Leydig cells more clearly (blue arrow). Scale bar length: 30 µm. (**IIA**): Section of testes from group 2 showing seminiferous tubules with irregular epithelium and loss of germ cells. Scale bar length: 200 µm. (**IIB**): Higher magnification of the same section, revealing degenerated germ cells (arrow). Scale bar length: 30 µm. (**IIC**): Section of testes from group 2 showing vascular congestion (Arrows). Scale bar length: 200 µm. (**IID**): Section of testis with group II showing sloughing of germinal epithelium (arrow). Scale bar length: 200 µm. (**IIIA**): Section of testes from group 3 showing normal architecture, having seminiferous tubules with active spermatogenesis and interstitial space with Leydig cells. Scale bar length: 200 µm. (**IIIB**): Higher magnification of the same section, revealing normal spermatogenesis and Leydig cells more clearly. Scale bar length: 30 µm. (**IVA**): Section of testes from group 4 showing partial restoration of tubule structure and hypo-spermatogenesis. Scale bar length: 200 µm. (**IVB**): Higher magnification of the same section, revealing the changes more clearly. Scale bar length: 30 µm. (**VA**): Section of testes from group 5 showing restoration of the architecture to near normal and intact germinal epithelium in majority of tubules and restoration of spermatogenesis. H.E. scale bar length: 200 µm. (**VB**): Higher magnification of the same section, revealing the changes more clearly. Scale bar length: 30 µm.

Microscopic examination of the testes revealed distinct group-wise changes (Figure 7). Groups 1 and 3 showed normal architecture of seminiferous tubules, active spermatogenesis, and intact interstitial tissue with healthy Leydig cells. In contrast, group 2 exhibited severe testicular damage, including disintegration of the seminiferous epithelium, loss of germinal cells, interstitial edema, vascular congestion, hemorrhage, and irregular tubules with sloughing of germinal epithelium. Group 4 showed partial recovery, with reduced congestion and edema, partial restoration of tubular structure, and hypo-spermatogenesis. Group 5 demonstrated near-complete restoration, displaying normal histological organization, intact germinal epithelium, and active spermatogenesis. The unedited and unmarked micrographs are provided in Figure S1.

## 4. Discussion

Because of its pleasant taste, MSG is now used as a flavoring agent in food. Many food and drug control agencies have approved MSG to be safe for human consumption without any specified dosage. However, because this food additive is widely used in various food ingredients, it may be abused unintentionally [25]. The public’s concerns about its transfer into the blood and raising brain glutamate levels, which would cause functional disruptions and affect other organs of the body, have been raised by its excessive inclusion in foods, necessitating a safety evaluation. This concern was initially raised a great deal and led to an extensive series of scientific studies examining this issue, mostly conducted in rodents [26]. Studies have particularly highlighted MG’s detrimental impact on the male reproductive system, even though it is associated with many negative effects on biological processes. The potential preventive benefits of eugenol against testicular damage caused by MG in male rats were investigated in this study.

Multiple studies have demonstrated that MSG exposure in rats leads to a pronounced imbalance in redox homeostasis, characterized by depletion of endogenous antioxidants such as reduced glutathione (GSH) and diminished activities of enzymatic scavengers like superoxide dismutase (SOD), concurrently with a marked rise in lipid peroxidation products (e.g., TBARS) [27]. Mechanistically, excessive glutamate from MSG may over-activate excitatory pathways (e.g., glutamate receptors), disturb mitochondrial function, and accelerate reactive oxygen species (ROS) generation [28]. This ROS overload overwhelms the antioxidant defense system, thereby oxidizing membrane lipids (increasing TBARS), damaging cellular proteins and DNA, and impairing antioxidant enzymes, leading to structural and functional damage in the liver, kidney, and other tissues [29]. A significant increase in the body weight of rats in group 2 vs. control was observed in this study, likely because it alters appetite-regulating pathways leading to increased food intake and energy storage. MSG exposure also causes hypothalamic damage, impairs satiety signals, and promotes obesity. Additionally, it also induces insulin resistance and enhanced fat deposition, together resulting in significant weight gain [30,31]. Testicular weight was decreased significantly in Group 2 compared with Groups 1 and 3, which significantly improved with eugenol. The reduction in testicular weight is because MSG induces oxidative stress and generates free radicals in testicular tissue, leading to cellular damage. It also disrupts the hypothalamic–pituitary–gonadal (HPG) axis, lowering testosterone production. These effects together impair spermatogenic cells and shrink testicular mass [29,32,33]. The variation in various hematological parameters observed in this study is because MSG induces oxidative stress and lipid peroxidation, which damage erythrocyte membranes and shorten their lifespan, leading to anemia [28]. MSG can also impair bone-marrow function and reduce erythropoiesis through disruption of the hypothalamic–pituitary axis and altered erythropoietin levels [34]. In contrast, the increase in TLC occurs due to inflammatory and immunological responses triggered by MSG-induced tissue injury, where the body elevates leukocyte production to counteract oxidative damage and inflammation [35]. Similarly, liver and kidney damage observed in this study is likely through the generation of oxidative stress, which leads to lipid peroxidation, mitochondrial dysfunction, and cellular injury in hepatic and renal tissues. Excessive free radicals overwhelm antioxidant defenses (SOD, CAT, and GSH), resulting in hepatocyte membrane damage and leakage of liver enzymes such as ALT and AST into the bloodstream. Similarly, oxidative and inflammatory injury to renal tubular cells impairs filtration capacity, causing elevations in BUN and creatinine. Inflammation, apoptosis, and ionic imbalance further contribute to structural and functional deterioration of both organs [27,28,34]. MSG exposure lowers serum testosterone in rats through several linked mechanisms. It generates oxidative stress in testicular tissue that damages Leydig cells and steroidogenic enzymes. Secondly, it disrupts the hypothalamic–pituitary–gonadal (HPG) axis via hypothalamic injury, further suppressing Leydig-cell testosterone synthesis. Also, MSG-triggered inflammation and apoptosis in the testis reduce viable steroidogenic cell mass and down-regulate steroidogenic proteins, together producing the observed fall in circulating testosterone [36,37,38].

The findings of the present study demonstrate that eugenol confers significant protective effects against monosodium glutamate (MSG)-induced toxicity in rats, highlighting its potential as a natural therapeutic agent. MSG administration is known to trigger oxidative stress, disrupt antioxidant defense systems, and induce tissue injury through excessive free radical generation and lipid peroxidation. In contrast, eugenol, an active phenolic constituent of clove oil, exhibits strong antioxidant, anti-inflammatory, and membrane-stabilizing properties, which may counteract these deleterious effects [13,16]. Findings from recent in vivo reproductive-toxicity models support the present results. Eugenol was reported to partially restore testicular histology, reduce oxidative and nitrosative markers, modulate apoptosis/autophagy markers, and normalize key signaling pathways in toxin-challenged rats. Such pathway modulation likely underlies recovery of serum testosterone and improvements in haemato-biochemical profiles following eugenol administration [17].

Monosodium glutamate (MSG) administration produced clear and reproducible testicular lesions in Wistar rats, characterized by disorganization and atrophy of the seminiferous epithelium, loss or exfoliation of spermatogenic cells (particularly spermatocytes and spermatids), tubular shrinkage/degeneration, and occasional Leydig-cell alterations, changes that are consistent with impaired spermatogenesis and reduced fertility [33]. These structural disruptions co-occur with biochemical evidence of oxidative stress in testicular tissue and with activation of cell-death pathways. Such a combination provides a parsimonious mechanistic explanation for the histological picture and for reported declines in sperm counts and serum testosterone after chronic MSG exposure [33]. It is important to note that the literature also reports dose-dependent and context-dependent effects of eugenol on male reproductive endpoints. Some studies observed reductions in serum testosterone and changes in sperm parameters at certain doses or regimens, indicating that eugenol’s net reproductive effects can vary with dose, treatment duration, and experimental conditions. The protective effects of eugenol observed in the present study may be attributed to its well-documented antioxidant and cytoprotective properties. Eugenol acts as a potent free radical scavenger, inhibits lipid peroxidation, and enhances endogenous antioxidant defenses such as superoxide dismutase and glutathione, thereby counteracting MSG-induced oxidative stress in testicular tissue [14,15,16]. In addition, eugenol has been shown to stabilize cellular membranes, modulate inflammatory signaling pathways, and preserve mitochondrial integrity, which collectively contribute to the maintenance of seminiferous tubule architecture and Leydig-cell steroidogenic activity [13]. Recent experimental studies further demonstrate that eugenol attenuates toxin-induced reproductive damage by suppressing oxidative stress-mediated apoptosis and inflammation while restoring hormonal balance and antioxidant enzyme activity [17]. These mechanistic findings from previous reports strongly support the protective effects observed in the present study against MSG-induced testicular toxicity. Therefore, while our data indicate a protective/hepatoprotective and testis-sparing role of eugenol against MSG-induced oxidative injury, careful dose selection and reporting of exposure timing are essential when extrapolating these results [39].

Although eugenol treatment resulted in significant improvement in most MSG-induced alterations, it is important to note that the recovery was not complete for all evaluated parameters. Several biochemical, hormonal, and antioxidant indices in the eugenol-treated groups remained modestly different from control values, indicating partial rather than full restoration. These findings suggest that eugenol primarily exerts a protective and ameliorative effect by attenuating the severity of MSG-induced toxicity, rather than completely reversing the damage. Such partial recovery may reflect the high experimental dose of MSG used, the limited duration of treatment, or the multifactorial nature of MSG-induced tissue injury, and underscores the need for cautious interpretation and further investigation. A limitation of the present study is the absence of direct sperm quality assessments (sperm count, motility, and morphology); however, the detailed histopathological evaluation of seminiferous tubules, antioxidant status, and serum testosterone levels provides reliable indirect evidence of spermatogenic impairment and recovery.

## 5. Conclusions

The present study demonstrates that oral administration of monosodium glutamate induces marked hematological, biochemical, hormonal, oxidative, and histopathological alterations in the testes of male Wistar rats, confirming its potential to impair male reproductive health under the experimental conditions employed. Co-administration of eugenol resulted in a significant, dose-dependent amelioration of these MSG-induced changes, as evidenced by the partial to near-complete restoration of antioxidant status, testicular architecture, and associated systemic parameters, particularly at the higher dose tested. These findings suggest that eugenol may mitigate MSG-induced reproductive toxicity primarily through its antioxidant and cytoprotective properties. Differences in species, dose, duration of exposure, and lack of functional fertility and molecular mechanistic assessments restrict direct extrapolation to humans. Further studies incorporating long-term exposure, mechanistic pathway analyses, reproductive performance outcomes, and clinical validation are warranted to better define the therapeutic potential and safety profile of eugenol. Overall, within the limitations of the present study, eugenol appears to be a promising natural compound for attenuating chemically induced testicular oxidative damage.

## Figures and Tables

**Table 1:** Body weights (Mean ± SE, g) of rats at weekly intervals in different experimental groups (n = 6). Group I: Control (normal saline); Group II: MSG (2.5 g/kg b.w.); Group III: Eugenol (200 mg/kg b.w.); Group IV: MSG (2.5 g/kg) + Eugenol (100 mg/kg); Group V: MSG (2.5 g/kg) + Eugenol (200 mg/kg). Data were analyzed using one-way ANOVA followed by Tukey’s multiple comparison test. Differences were considered statistically significant at *p* < 0.05. The superscript letters (a–c) in Table 1 denote statistical comparisons among groups at the same time point. Values sharing the same superscript letter are not significantly different from each other, whereas values with different superscript letters indicate a statistically significant difference (*p* < 0.05). Specifically, “a” indicates no significant difference compared with Group 1; “b” indicates a statistically significant difference compared with Group 1; and “c” indicates a statistically significant difference compared with both Groups 1 and 2.

Group	0 Day	7th Day	14th Day	21st Day	28th Day
Group-1	175.1 ± 2.92 ^a^	183.7 ± 3.19 ^a^	189.4 ± 4.35 ^a^	199.1 ± 4.05 ^a^	206.3 ± 4.19 ^a^
Group-2	175.7 ± 3.95 ^a^	184.6 ± 2.71 ^a^	195.9 ± 2.31 ^b^	207.2 ± 1.95 ^b^	215.7 ± 1.98 ^b^
Group-3	171.7 ± 4.23 ^a^	179.7 ± 4.07 ^a^	187.9 ± 3.77 ^a^	198.1 ± 4.09 ^a^	206.6 ± 3.78 ^a^
Group-4	175.4 ± 2.07 ^a^	187.1 ± 2.25 ^ab^	194.2 ± 3.04 ^b^	206.1 ± 2.84 ^b^	215.8 ± 3.25 ^b^
Group-5	178.3 ± 1.77 ^a^	188.4 ± 1.68 ^ab^	199.9 ± 1.32 ^c^	206.1 ± 1.82 ^b^	220.5 ± 2.15 ^c^

## Data Availability

The original contributions presented in this study are included in the article/Supplementary Material. Further inquiries can be directed to the corresponding authors.

## Supplementary Materials

The following supporting information can be downloaded at: https://www.mdpi.com/article/10.3390/jox16010033/s1; Figure S1: Unedited and unmarked micrographs of Figure 7.

## References

[1] Pepper A.N., Sriaroon P., Glaum M.C. (2020). Additives and preservatives: Role in food allergy. J. Food Allergy.

[2] Anbarkeh F.R., Baradaran R., Ghandy N., Jalali M., Reza Nikravesh M., Soukhtanloo M. (2019). Effects of monosodium glutamate on apoptosis of germ cells in testicular tissue of adult rat: An experimental study. Int. J. Reprod. Biomed..

[3] Kurihara K. (2009). Glutamate: From discovery as a food flavor to role as a basic taste (umami). Am. J. Clin. Nutr..

[4] Petersen A.M., Louw J., Görgens J.F. (2024). Economic and environmental comparison of the monosodium glutamate (MSG) production processes from A-molasses in an integrated sugarcane biorefinery. Int. J. Chem. Eng..

[5] Geha R.S., Beiser A., Ren C., Patterson R., Greenberger P.A., Grammer L.C., Ditto A.M., Harris K.E., Shaughnessy M.A., Yarnold P.R. (2000). Review of alleged reaction to monosodium glutamate and outcome of a multicenter double-blind placebo-controlled study. J. Nutr..

[6] Banerjee A., Das D., Paul R., Roy S., Bhattacharjee A., Prasad S.K., Banerjee O., Mukherjee S., Maji B.K. (2020). Altered composition of high-lipid diet may generate reactive oxygen species by disturbing the balance of antioxidant and free radicals. J. Basic Clin. Physiol. Pharmacol..

[7] Jubaidi F.F., Mathialagan R.D., Noor M.M., Taib I.S., Budin S.B. (2019). Monosodium glutamate daily oral supplementation: Study of its effects on male reproductive system on rat model. Syst. Biol. Reprod. Med..

[8] Aitken R.J., Gibb Z., Baker M.A., Drevet J., Gharagozloo P. (2016). Causes and consequences of oxidative stress in spermatozoa. Reprod. Fertil. Dev..

[9] Hamza R.Z., Diab A.E. (2020). Testicular protective effects of selenium nanoparticles against testicular toxicity. Toxicol. Rep..

[10] Hajihasani M.M., Soheili V., Zirak M.R., Sahebkar A., Shakeri A. (2020). Natural products as safeguards against monosodium glutamate-induced toxicity. Iran. J. Basic Med. Sci..

[11] Sharma A. (2015). Monosodium glutamate-induced oxidative kidney damage and possible mechanisms: A mini-review. J. Biomed. Sci..

[12] Khalil A.A., Rahman U., Khan M.R., Saha A., Mehmood T., Khan M. (2017). Essential oil eugenol: Sources, extraction, nutraceutical perspectives. RSC Adv..

[13] Ulanowska M., Olas B. (2021). Biological Properties and Prospects for the Application of Eugenol—A Review. Int. J. Mol. Sci..

[14] Ito M., Murakami K., Yoshino M. (2005). Antioxidant action of eugenol compounds: Role of metal ion in the inhibition of lipid peroxidation. Food Chem. Toxicol..

[15] Oroojan A.A., Chenani N., Anaam M. (2020). Antioxidant effects of eugenol on oxidative stress induced by hydrogen peroxide in islets of Langerhans isolated from male mouse. Int. J. Hepatol..

[16] Adefegha S.A., Oyeleye S.I., Okeke B.M., Oboh G. (2018). Influence of eugenol on oxidative stress biomarkers in the liver of carrageenan-induced arthritis rats. J. Basic. Clin. Physiol. Pharmacol..

[17] Saleh D.O., Baraka S.M., Jaleel G.A.A., Hassan A., Ahmed Farid O.A. (2024). Eugenol alleviates acrylamide-induced rat testicular toxicity by modulating AMPK/p-AKT/mTOR signaling pathway and blood–testis barrier remodeling. Sci. Rep..

[18] Zari A.T., Zari T.A., Hakeem K.R. (2021). Anticancer Properties of Eugenol: A Review. Molecules.

[19] National Center for Biotechnology Information (NCBI) (2024). PubChem Compound Summary for Monosodium Glutamate.

[20] Ellman G.L. (1959). Tissue sulfhydryl groups. Arch. Biochem. Biophys..

[21] Kakkar P., Das B., Viswanathan P.N. (1984). A modified spectrophotometric assay of superoxide dismutase. Indian J. Biochem. Biophys..

[22] Ohkawa H., Ohishi N., Yagi K. (1979). Assay for lipid peroxides in animal tissues by thiobarbituric acid reaction. Anal. Biochem..

[23] Luna G.L.H.T. (1968). Manual of Histological and Special Staining Techniques.

[24] Snedecor G.W., Cochran W.G. (1989). Statistical Methods.

[25] Hamza R.Z., AL-Harbi M.S. (2014). Monosodium glutamate induced testicular toxicity and the possible ameliorative role of vitamin E or selenium in male rats. Toxicol. Rep..

[26] Gong S.L., Xia F.Q., Wei J., Li X.Y., Sun T.H., Lu Z., Liu S.Z. (1995). Harmful effects of MSG on function of the hypothalamus–pituitary–target gland system. Biomed. Environ. Sci..

[27] Abdou H.M., El-Gendy A.H., Aly R.G., Abouzied M.M., Eltahir H.M., Al Thagfan S.S., Eweda S.M. (2025). Evaluation of the Effects of Monosodium Glutamate Overconsumption on the Functions of the Liver, Kidney, and Heart of Male Rats: The Involvement of Dyslipidemia, Oxidative Stress, and Inflammatory Responses. J. Xenobiot..

[28] Farombi E.O., Onyema O.O. (2006). Monosodium glutamate-induced oxidative damage and genotoxicity in the rat: Modulatory role of vitamin C, vitamin E and quercetin. Hum. Exp. Toxicol..

[29] Askar M.E., Abdel-Maksoud Y.T., Shaheen M.A., Eissa R.G. (2025). Ameliorating monosodium glutamate-induced testicular dysfunction by modulating steroidogenesis, oxidative stress, inflammation, and apoptosis: Therapeutic role of hesperidin. Biochem. Biophys. Res. Commun..

[30] Bautista H.R.J., Mahmoud A.M., Königsberg M., López Díaz Guerrero N.E. (2019). Obesity: Pathophysiology, monosodium glutamate-induced model and anti-obesity medicinal plants. Biomed. Pharmacother..

[31] Kahe K., Laferrere B., Castellanos F.X., Zhang Y., Mozaffarian D. (2025). Monosodium glutamate: A hidden risk factor for obesity?. Obes. Rev..

[32] Abdul-Hamid M., Galaly S.R., Ahmed R.R., Hamdallah H.M. (2021). Histopathological and biochemical effect of quercetin on monosodium glutamate supplementation-induced testicular toxicity. Beni-Suef Univ. J. Basic Appl. Sci..

[33] Kianifard D., Shoar S.M.M., Karkan M.F., Aly A. (2021). Effects of monosodium glutamate on testicular structural and functional alterations induced by quinine therapy in rat: An experimental study. Int. J. Reprod. Biomed..

[34] Emmanuel N.S., Yusuf T., Bako I.G., Malgwi I.S., Eze E.D., Ali Z., Aliyu M. (2024). Hematological changes, oxidative stress assessment, and dysregulation of aquaporin-3 channel, prolactin, and oxytocin receptors in kidneys of lactating Wistar rats treated with monosodium glutamate. Naunyn Schmiedebergs Arch. Pharmacol..

[35] Motwadie M.E., Hashem M.M., Abo-El-Sooud K., Abd-Elhakim Y.M., El-Metwally A.E., Ali H.A. (2021). Modulation of immune functions, inflammatory response, and cytokine production following long-term oral exposure to three food additives; thiabendazole, monosodium glutamate, and brilliant blue in rats. Int. Immunopharmacol..

[36] Kayode O.T., Rotimi D.E., Kayode A.A.A., Olaolu T.D., Adeyemi O.S. (2020). Monosodium glutamate (MSG)-induced male reproductive dysfunction: A mini review. Toxics.

[37] Koohpeyma F., Gholizadeh F., Hafezi H., Hajiaghayi M., Siri M., Allahyari S., Maleki M.H., Asmarian N., Bayat E., Dastghaib S. (2022). The protective effect of L-carnitine on testosterone synthesis pathway, and spermatogenesis in monosodium glutamate-induced rats. BMC Complement. Med. Ther..

[38] Oluwole D.T., Ebiwonjumi O.S., Ajayi L.O., Alabi O.D., Amos V., Akanbi G., Adeyemi W.J., Ajayi A.F. (2024). Disruptive consequences of monosodium glutamate on male reproductive function: A review. Curr. Res. Toxicol..

[39] Carvalho R.P.R., Lima G.D.A., Ribeiro F.C.D., Ervilha L.O.G., Oliveira E.L., Viana A.G.A., Machado-Neves M. (2022). Eugenol reduces serum testosterone levels and sperm viability in adult Wistar rats. Reprod. Toxicol..

