# Supplementary figures and images for: Protective Effects of Eugenol Against Monosodium Glutamate-Induced Reproductive Toxicity in Male Wistar Rats
Source: J Xenobiot. 2026 Feb 13;16(1):33. doi: 10.3390/jox16010033 (PMC12921986; doi:10.3390/jox16010033)

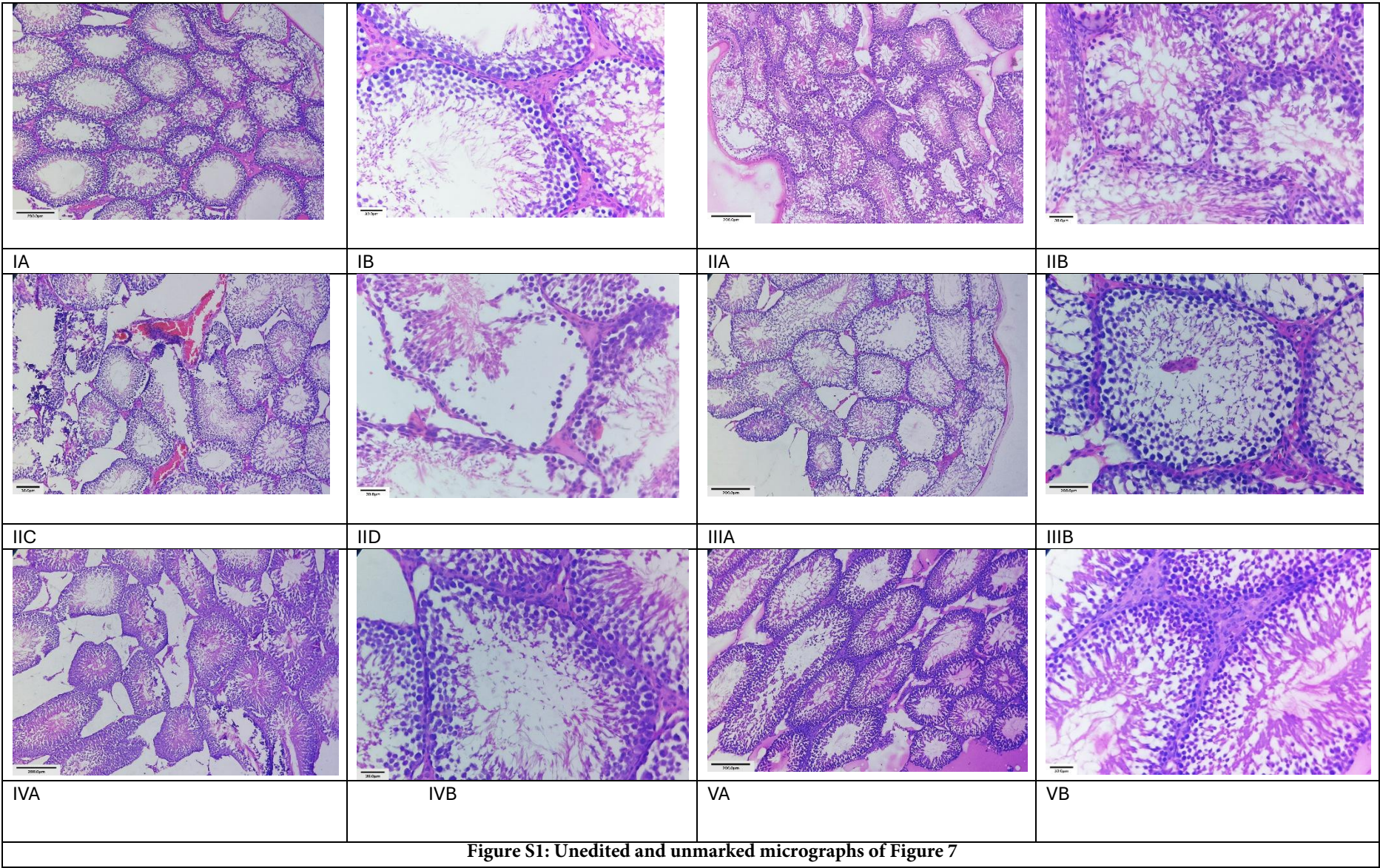

Supplement: Supplementary file 1 [file jox-16-00033-s001.zip › jox-4104432-supplementary.pdf]
